# The provider perspective: investigating the effect of the Electronic Patient-Reported Outcome (ePRO) mobile application and portal on primary care provider workflow

**DOI:** 10.1017/S1463423617000573

**Published:** 2017-09-13

**Authors:** Parminder K. Hans, Carolyn Steele Gray, Ashlinder Gill, James Tiessen

**Affiliations:** 1 Bridgepoint Collaboratory, Lunenfeld-Tanenbaum Research Institute, Sinai Health System, Toronto, ON, Canada; 2 Institute for Health Policy, Management and Evaluation, Dalla Lana School of Public Health, University of Toronto, Toronto, ON, Canada; 3 School of Health Services Management, Ted Rogers School of Management, Ryerson University, Toronto, ON, Canada

**Keywords:** complex care, mHealth, multi-morbidity, primary care provider, resistance, workflow

## Abstract

**Aim:**

This qualitative study investigates how the Electronic Patient-Reported Outcome (ePRO) mobile application and portal system, designed to capture patient-reported measures to support self-management, affected primary care provider workflows.

**Background:**

The Canadian health system is facing an ageing population that is living with chronic disease. Disruptive innovations like mobile health technologies can help to support health system transformation needed to better meet the multifaceted needs of the complex care patient. However, there are challenges with implementing these technologies in primary care settings, in particular the effect on primary care provider workflows.

**Methods:**

Over a six-week period interdisciplinary primary care providers (*n*=6) and their complex care patients (*n*=12), used the ePRO mobile application and portal to collaboratively goal-set, manage care plans, and support self-management using patient-reported measures. Secondary thematic analysis of focus groups, training sessions, and issue tracker reports captured user experiences at a Toronto area Family Health Team from October 2014 to January 2015.

**Findings:**

Key issues raised by providers included: liability concerns associated with remote monitoring, increased documentation activities due to a lack of interoperability between the app and the electronic patient record, increased provider anxiety with regard to the potential for the app to disrupt and infringe upon appointment time, and increased demands for patient engagement. Primary care providers reported the app helped to focus care plans and to begin a collaborative conversation on goal-setting. However, throughout our investigation we found a high level of provider resistance evidenced by consistent attempts to shift the app towards fitting with existing workflows rather than adapting much of their behaviour. As health systems seek innovative and disruptive models to better serve this complex patient population, provider change resistance will need to be addressed. New models and technologies cannot be disruptive in an environment that is resisting change.

## Introduction

The medium in which healthcare is delivered is often a reflection of the times it serves. Increasing healthcare costs, an ageing population, and increasing prevalence of chronic illness and multi-morbidity, has resulted in healthcare systems, organizations, and providers seeking new models and innovations in care delivery. Health technologies in particular are gaining prevalence. The latest subset of the electronic healthcare revolution, mobile health commonly referred to as mHealth, sees mobile-based platforms and applications (apps) deliver health information over the internet and into the palm of our hand (Martinez, 2011; West, 2012; Hamine *et al*., 2015). Currently 165 000 health apps designed to help users monitor health, fitness, and well-being are available (Morgan and Agee, 2011; *The Economist*, 2016) with downloads projected at 1.7 billion by 2017 (*The Economist*, 2016) and demand increasing year-over-year (Leijdekkers and Gay, 2012; Elias, 2015; *The Economist*, 2016).

This recent trend toward readily available mHealth solutions is forcing health professionals to alter current care practices to adapt to the digitally engaged patient (Lupton, 2006; Morgan and Agee, 2011), and in particular the growing number of patients requiring chronic disease management. The Canadian Community Health Survey indicates almost 80% of Ontarians over the age of 45 suffer from chronic disease, of which ~70% suffer multi-morbidity, the presence of two or more chronic diseases that require ongoing care coordination, increased supports, and resources (Tsasis and Bains, 2008; OECD, 2011; Schaink *et al*., 2012; Steele Gray *et al*., 2014). But, is there a case for mHealth to tackle chronic disease management? A recent survey (Levy, 2012) of 1027 patients worldwide with diverse health conditions reported 48% of respondents feeling ‘mHealth will change the way they manage their chronic illnesses and medications’ (Morgan and Agee, 2011: 5). Although, the potential cost savings generated from mHealth solutions in Canada have yet to be reported, West (2012) indicates remote monitoring has the potential to save the United States $197 billon over the next 25 years, with the greatest benefit expected in the area of chronic disease management. Recent literature suggests mobile health technology is an ideal tool to manage chronic disease through its ability to: ‘submit and process data, automate messaging, and provide consultations as needed in a discreet, timely, and personalized manner’ (Mechael *et al*., 2010: 18), and plays a vital role in ‘coordinat[ing] and integrat[ing] care across a complex health care system’ (Martin, 2012: 937).

Better care management afforded through mHealth allows providers to work more efficiently when access to patient information is readily available (Varshney, 2007; Martinez, 2011), and with the potential to improve care delivery, management, and patient outcomes (Levin, 2011; Morgan and Agee, 2011), is it any wonder why Intel believes 50% of healthcare could be provided through a brick-less clinic in the next 10 years? (Morgan and Agee, 2011). However, promises of improved system productivity, efficiency, and care quality (Yu *et al*., 2006) may overextend the current capabilities of mHealth, as strong evidence to demonstrate improved health and healthcare as a result of mHealth is lacking (Martin, 2012; Walton, 2012). Healthcare continues to struggle with pairing innovative health information technologies (HIT) with existing organizational, technical, and clinical practice requirements (Ford *et al*., 2006; Cresswell and Sheikh, 2013); ‘while the growing popularity of mHealth is evident, its impact is not’ (Hamine *et al*., 2015: 2). Most apps are rarely if ever used (*The Economist*, 2016), move beyond the pilot stage, or provide best strategies for effective scale-up (Tomlinson *et al*., 2013). Add to this mix a poorly designed app that ignores human factors such as workflow and now the app becomes altogether ineffective (Levin, 2011; Steinhubl *et al*., 2013).

### mHealth effect on primary care provider workflow

Sustainable adoption of mHealth applications that meet the changing health needs of patients may only occur if providers are: (1) willing to redesign their workflow practices, and (2) accept the integration of disruptive technology that will inevitably alter care practices. A summary of key aspects of adoption detailed in the literature are described in this section.

**Figure 1:**
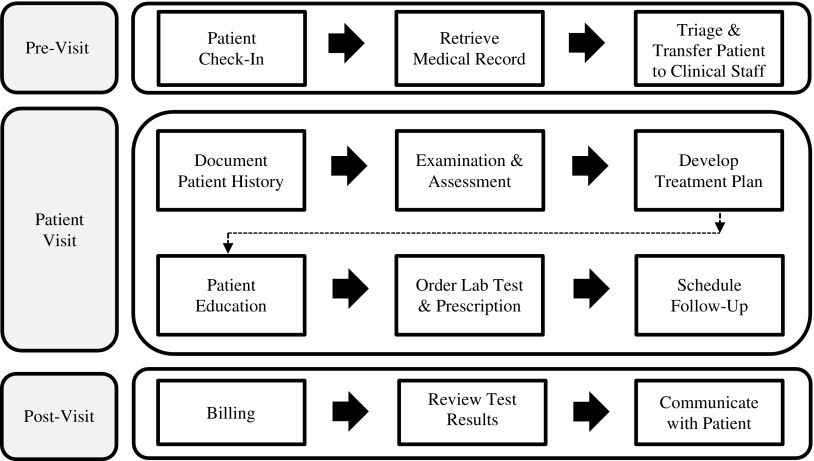
Typical primary care provider workflow: pre-visit, patient visit, and post-visit. Clinical Workflow Process Diagram adapted from Bowens *et al*. (2010) and Lee and Shartzer (2005: 1–2).

First, the introduction of new technologies into the workplace not only augments work routines, but in fact, reorganizes them (Medina-Mora *et al*., 1992; Walton, 2012; Li *et al*., 2013). Increases in documentation practices, suspicious or unreliable data entries, impaired patient visits that shift the conversation towards the technology rather than the patient, and tech literacy can all effect workflows and mHealth adoption (Yu *et al*., 2006; Zheng *et al*., 2010; Alsos *et al*., 2012; Leijdekkers and Gay, 2012; Li *et al*., 2013). Clinical workflow is defined as tasks which healthcare professionals are required to perform to generate outcomes to promote and provide healthcare (Figure 1), and should be thoroughly investigated before the adoption of new technology (Lee and Shartzer, 2005; Campbell *et al*., 2009; Bowens *et al*., 2010; Zheng *et al*., 2010).

Second, the challenge to overcome provider reluctance in adopting mHealth applications should be studied. Provider perceptions and attitudes toward mHealth present a larger implementation barrier than the technology itself (Mirza *et al*., 2008). If health professionals are resistant to effectively incorporate disruptive change, or do not possess the necessary attributes such as growth-orientation, motivation, efficacy, or adaptability, sustained innovation is unlikely (Lehman *et al*., 2002; Li *et al*., 2013). Ward *et al*. (2008) coins the term ‘resistive compliance’ to reveal the ways providers work around the introduction of new electronic health innovations without altogether rejecting its use (ie, minimize interaction with new technology, label technology as impersonal or useless).

But nonetheless the appetite for mHealth exists. Constant workflow fluctuations feed provider demand for mHealth solutions capable of storing, transmitting, and capturing health information on-the-go (Yu and Yu, 2004; Yu *et al*., 2006). The question now becomes are we simply shifting practices to embrace digitization without adequately examining if it is really beneficial and safe to do so? (Lupton, 2006). This qualitative study investigates how one mobile app piloted in a primary healthcare team environment impacts provider workflow and whether providers resist its adoption as they care for their patients with complex care needs.

### The App: electronic Patient-Reported Outcome (ePRO) mobile application and portal

In 2013 the Health System Performance Research Network and the Bridgepoint Collaboratory began their multi-phase multi-method trial to develop an innovative patient-centred app. Earlier study phases brought together primary care providers, complex care patients, caregivers, content experts, and information technology developers to discuss key app features that could improve self-management and care delivery for patients with complex care needs and their primary care providers. By engaging the end user throughout the development process our priority was to produce a mobile app that was both useful and functional to providers and patients. Through an iterative development process the Electronic Patient-Reported Outcome mobile application and portal (hereinafter ePRO or app) was created to support patient self-management and guide care plans. The app allows patients and providers to:

(1) Collaboratively create health goals with attached monitoring protocols.
(2) Foster a continued relationship as providers are able to view real-time patient monitoring and health goal progress. Although providers are not alerted when patient entries are reported, providers have the ability to look up patient reports at any time via the ePRO portal. In-app messaging is not possible although patients could send comments viewable to providers via the free text feature.
(3) Adjust health goals and monitor protocols to adapt to ongoing patient needs.
(4) Capture and report standardized outcome measures to aid shared decision-making and care planning activities including:

∙ Patient-Reported Outcomes Measurement Information System (PROMIS) Global Health Scale
∙ PROMIS Pain Interference Scale (Short Form 8a)
∙ PROMIS Health Assessment Questionnaire (HAQ)
∙ Generalized Anxiety Disorder Scale (GAD-7)
∙ Patient Health Questionnaire (PHQ-9)

(5) Hospital alert: patient initiated message informs provider of their discharge from hospital.

The app, preloaded onto Samsung Core smartphones complete with 3G data coverage, was provided to patients for the duration of the study. Providers accessed the desktop version of the app via a portal system. Details regarding development of the app as well as findings from a larger usability assessment have been published elsewhere (Steele Gray *et al*., 2014; 2016a; 2016b).

## Material and methods

Findings presented in this work were extracted from data collected during the usability assessment conducted through a six-week pilot study. Qualitative methodology guided data collection and analysis. All ethical requirements were adhered to throughout the study and consent was received from participants before the initiation of study activities.

### Participants

The pilot study was conducted at a Toronto area Family Health Team (FHT) over the period of October 2014 to January 2015. In total, six interdisciplinary healthcare professionals (P1–6) served as the provider sample, although only five providers actively used and monitored patient reporting via the app. All providers who care for complex care patients at the FHT were invited to participate in our study. Interested patients with complex care needs [defined as either: two or more chronic conditions with (1) emergency department visit or hospitalization in the previous six months or 10 plus primary care visits in the past year, and/or (2) identified by providers or self-identified as having complex care needs], who were available during the study period were invited to participate. In total 12 complex care patients served as the patient sample (PT1–12).

For the purpose of the study, patients and providers were required to meet on two separate occasions. At Week 1 to set health goals and monitoring protocols, and at Week 6 to discuss their experience in using the app. Both patients and providers were free to meet outside of these scheduled visits as a part of routine care, although this was not mandatory.

### Data analysis

Secondary data analysis of provider and patient experience was conducted. Data were thematically analyzed and extracted from four sources (Table 1). Provider and patient focus groups were semi-structured and consisted of preset open-ended questions (Table 2). Two researchers (A.G. and P.K.H.) independently reviewed the data and began open coding (Elo and Kyngäs, 2008). As is consistent with qualitative thematic analysis methods, multiple readings of the data sets allowed the researchers to orient themselves to the material before categorizing portions of the texts (Gallicano, 2013; Vaismoradi *et al.*, 2013). Researchers independently searched, refined, and grouped initial concepts and generated tentative codes for the emergent themes. The tentative themes structured the preliminary codebooks.

**Table 1:** Data source for qualitative analysis

**Training notes (TN)**	
Definition	App and portal training co-facilitated by research team and app developer, QoC Health
Data source	Field notes recorded by research team member (AIK)
Frequency and duration	27 and 30 October 2014; 60-min sessions
**Patient focus group (PTFG)**	
Definition	Semi-structured guided discussion moderated by CSG
Data source	12 complex chronic care patients (PT1–12)
Frequency and duration	18 December 2014; 75 min
**Provider focus group (PFG)**	
Definition	Semi-structured guided discussion moderated by CSG
Data source	6 interdisciplinary healthcare providers (P1–6)
Frequency and duration	22 January 2015; 60 min
**Issue tracker report (TR)**	
Definition	Patient and provider reported usability experiences (ie, troubleshooting and technical concerns)
Data source	Patients (PT1–12) and providers (P1–6)
Frequency and duration	October 2014–January 2015; inclusive

**Table 2:** Focus group semi-structured scripts

**Provider**
1. How did you use the app?
Prompt: How did it work into your day-to-day workflow?
2. Did this app help you manage the care of patients?
Prompt: What’s missing or could be added?
3. Is the app easy to use and understand in terms of how the information is presented and accessed?
4. Are there other ways that we could gather similar information from you (ie, linking to other apps or portals that are used by providers)?
**Patient**
1. How did you use the app?
2. Did the app adequately capture the issues of importance to you?
Prompt: What’s missing or could be added?
3. Is the app easy to use in terms of (1) the wording of the questions and (2) how you enter information?
4. Are there other ways that we could gather similar information from you? (ie, linking to other apps that you already use)?

Following the creation of two independent codebooks, the researchers collaboratively and iteratively worked through the themes and developed a single unified codebook that contained full definitions, boundaries of when to use the code, and examples of the codes. In addition to the overarching themes, subthemes were defined within each code to provide a fuller representation of that category. Participants, own words were used ‘to guide the construction of codes and their definitions’, thus allowing the data sets to guide analysis and reduce interpretation bias (MacQueen *et al*., 1998: 33). The consolidated codebook was reviewed by a third team member (CSG) who compared the codebook to data to validate themes and subthemes.

The consolidated codebook was used by P.K.H. and A.G. to each independently code the data sets. Intercoder agreement was assessed following the independent review of the data, discrepancies were noted and the codebook was revised as necessary. All data sets were equally weighted and coded line-by-line according to the finalized and agreed-upon codebook, prior to using QSR NVivo 10 software to organize data (QSR International Pty Ltd, 2016). Through this process the research team identified five themes and 14 subthemes.

Data validation was achieved through investigator and methodological triangulation that is commonly employed in social science research to increase topic understanding (Farmer *et al*., 2006). Investigator triangulation (the involvement of two or more researchers) and methodological triangulation (multiple data sources: focus groups, training sessions, and issue tracker reports) increased the likelihood of credible and dependable analysis as interpretations were cross-checked over a variety of data sources and perspectives (Guba, 1981; Krefting, 1991; Farmer *et al*., 2006; Yeasmin and Rahman, 2012). In addition, member checking was performed to further establish data credibility and validity, reduce the chances of data misrepresentation, and to ensure the research team accurately captured participant experiences (Lincoln and Guba, 1985; Krefting, 1991; Fereday and Muir-Cochrane, 2006). No discrepancies in data were noted.

## Results

Examination of the themes and subthemes illuminated how the app affected provider workflow while preparing for the patient appointment (pre-visit), interacting with the patient (patient visit), and remotely monitoring patient feedback (post-visit). These themes and subthemes were mapped onto primary care provider workflow activities; core themes around each workflow were then explored to determine what key issues arose at each visit stage and in particular if resistance in adopting the app were observed (Table 3). In addition, provider and patient demographics and protocol monitoring reports contextualized app usage (Tables 4 and 5, respectively).

**Table 3:** Theme occurrences across provider workflow: pre-visit, patient visit, and post-visit

Themes	Pre-visit	Patient visit	Post-visit
App use			
Provider app use	x	x	x
Align existing practice workflow with app	x	x	x
Patient monitoring	x	x	x
Barriers to adoption	x	x	x
Point-of-care		x	
Patient experience			
Patient readiness		x	x
Goal-setting		x	
Content			
Questions	x	x	x
Matching patient goals to preset categories		x	
Comprehensiveness		x	
Functionality			
App interface	x		
Errors		x	
Improvement			
Modifications			x
New addition		x	x

**Table 4:** Provider demographics and protocol monitoring reports

Primary care providers (*n*=6)	Number of patients	Number of care plans monitored	Number of app logins
Clinical (4)			
Primary care physician (MD)^a^			
Nurse			
Case manager (RN)	2	2	5
Educator (RN)	2	4	15
Practitioner (PHCNP)	3	4	12
Allied health (2)			
Dietitian educator (RD)	1	4	4
Social worker (RSW)	2	5	15
Total	10^b^	19	51
Mean	2.0	3.8	10.2

a Clinician lead supported study management; did not actively participate in intervention.

b A total of 12 patients consented to participate; however, two patients withdrew due to worsening health.

**Table 5:** Patient demographics and protocol monitoring reports

Participants (*n*=12)	*n* (%)	Chronic condition	*n* (%)
Female	6 (50.0)	Arthritis	2 (16.7)
Male	6 (50.0)	Cardiovascular	3 (25.0)
		Chronic pain	5 (41.7)
Age (years)		Diabetes	5 (41.7)
35–45	2 (16.7)	Mental health	10 (83.3)
46–55	3 (25.0)	Obesity	2 (16.7)
56–65	4 (33.3)	Renal failure	2 (16.7)
65+	3 (25.0)	Respiratory (COPD)	1 (8.3)
		Other	2 (16.7)
Mean	56.3	Mean	2.75
**Goal and protocol monitoring**			
Goal theme	Total protocol questions	Unique completions^a^	Mean time to complete report (min)^b^
Diet	3	9	1.0
Hospital alert	2	1	–
Mobility	25	3	10.3
Mood and memory	8	70	4.1
Pain	8	53	4.7
Physical health	9	76	2.1

a Unique completions: frequency of times patients completed full report of protocol questions.

b Outliers removed (min 683, max 1428 min). If patient left survey in the middle of reporting, system continued to count time until protocol completion.

Source: Steele Gray *et al*. (2016a) for complete patient usage data.

### Pre-visit

Providers reported that the app presented an additional resource that they could leverage to quickly orient themselves to their patients’ well-being: ‘*I did look at it before they came in…more out of curiosity to see like if there was some data there*’ (PFG, P4). However, the effectiveness of that resource was limited due to how information was presented on the app. The raw unfiltered data were unhelpful to one provider, as it confused more than assisted them in preparing for their visit; ‘*thinking about me and what my day looks like…show me a graph. Because all that other stuff, it might be good to have in there…[but it] overloads me*’ (PFG, P6). Although the app was designed with the intention to provide a simplified user platform to allow for easy viewing, to view aggregated data was not possible. Despite this challenge, at least one provider saw the potential for the tool to streamline their upcoming visit workflow:

‘*But I think the point is it has to fit into workflow, right. And so maybe it saves time; especially on certain patients… You have a whole lot of data that is very efficient. And then you can springboard as opposed to having them report to you … we’re almost [on] the same page from go time*’.

(PFG, P3)

Workflow considerations by one provider influenced the selection of patients to be recruited into the study. The provider selected patients, not necessarily because that patient would most benefit from the goal-setting application, but because choosing a patient that was familiar to them in terms of medical condition and needs, would make incorporating the app easier into their pre-visit routine: ‘*Because what I did was I chose people that I see weekly anyway and I didn’t have to have any extra work or whatever*’ (PFG, P6)

### Patient visit

During the visit the app seemed to encourage both patients and providers to collaboratively discuss health goals and effectively move through the layers of therapy to goal-set, which one provider found particularly rewarding:

‘*And it’s important but it’s hard to prioritize and say all we’re going to talk about today for the next half hour is this goal-setting. So that was helpful*…*To be able to talk about this with [the patient], it was like, oh, thank God I can help set a goal. Because that’s also about my job satisfaction, which I don’t always get because you’re always just kind of dealing with the stuff that hits you*’.

(PFG, P6)

Before setting the health goal, goal priority and the patient’s confidence in achieving the goal were assessed and recorded in the app. One provider discussed how the data were used during the visit to kick-start a conversation concerning self-management, and additionally offered their insight into how the app could be useful moving forward:

‘*So it was interesting to see where there were good days and asking a little bit more about that versus maybe some of the bad days and what was going on there*’.

(PFG, P4)

[Provider speaking of the potential of the app] ‘*there is going to be good periods, there’s going to be bad periods. And okay, it looks like you might be entering a bad period. And when you are, you recognize that. And now you’ve got coping strategies and mechanisms in place to deal with it*’

(PFG, P4)

Interoperability between the app and the electronic medical record (EMR) was found to increase documentation workload during the patient visit. All providers agreed with the sentiments voiced by one provider:

‘*In going with it has to be tailored to work flow, I would like that the visit could be somehow linked into our EMR. Because I was documenting, right. So I was like dealing with that template and setting the goal. But then that doesn’t document it in the patient’s chart*… *So it’s double work*’.

(PFG, P4)

Indeed this type of double documentation work can occur when adopting new HIT solutions (Li *et al*., 2013). Likewise, providers reported feeling the app did not support all care management activities. Resources were needed beyond what the app could provide to ensure patient care needs were met:

‘*[ePRO] wasn’t very comprehensive… if I wanted to give them resources or websites or handouts, that’s all separate…there wasn’t a lot of draw for them to go back to the [app]because of all of these external resources*’.

(PFG, P1)

The app additionally did not fit with the usual way goal-setting was done as part of their existing care management process. Providers reported they typically use the S.M.A.R.T (Specific, Measurable, Attainable, Realistic, Timed) goal template that was not available on the app. Thinking of how other providers were discussing this issue, one provider stated:

‘*The intention was good but the [app] didn’t fit into the existing workflow. It sounds like [the other providers] are clearly saying we use a SMART goal template, that’s how they do it. Now they’re kind of doing it a bit differently. And if it was in the workflow, probably it would have been perceived as a little bit more helpful*’.

(PFG, P3)

In addition, when asked about the usefulness of patient reports using the validated standardized outcome measures available in the app, participants agreed with the sentiments voiced by one provider: ‘*I didn’t find those questions all that helpful. It might be like a pre and post but maybe not in the middle*’ (PFG, P4).

The effect of mHealth interference on provider–patient visit time was also discussed. Two providers voiced their concerns with the app and its potential disruptive nature during the training sessions, explicitly questioning: ‘*whether it was worth sacrificing valuable patient and provider time*’ (TR, AIK) and even suggested using the app: ‘*might end up dominating the session*’ (TR, AIK).

### Post-visit

The app was designed with the intention to foster a more collaborative relationship between the provider and patient in terms of patient-centred goal-setting; however, this practice raised care expectations for patients:

‘*I’ve entered the questions religiously and I am in pain, and it’s every day for 10 days or two weeks or three weeks…, and you didn’t get any response. So then you’re going to say, well, no one cares about me*’.

(PTFG, PT12)

Providers noted that patients expressed the need for feedback but, as one provider indicated, incorporating the app into their daily work routines was not practical: ‘*But realistically in terms of workload or whatever, there’s no way. Even one patient, I bet I would not look at it, you know, every day*’ (PFG, P6). Two providers suggested automated push-messaging can be used to provide feedback to their patients at predetermined goal milestones:

‘*I think there’s some way, you can probably get acknowledgement or recognition for what you did. And it probably sends [the patient] some messages or something*’.

(PFG, P4)

Even though the app recorded goal-setting information patients found helpful: ‘…*I knew why I felt better one week and why I didn’t feel better the next week…*’ (PTFG, PT11), providers still questioned the importance of using the technology: ‘*maybe [the patient] gave you feedback saying that was really helpful, I don’t know. I didn’t think it was all that helpful. At least it wasn’t helpful from my perspective*’ (PFG, P4). Providers questioned whether the app would actually improve workflow functions or simply add another task. Multiple providers expressed their concerns with incorporating the ePRO into their daily visit routine:

‘*But it’s kind of nice I think for [the patient] to have that data in a place, to say, well, I didn’t accomplish these during that time period, and look at this. So it kind of gives a bigger picture and it makes more sense… But again, would we have not have figured that out otherwise?*’

(PFG, P2)

Providers found it difficult to remotely monitor their patients without the ability to directly observe them. Subjective data entries resulted in unnecessary safety checks, created extra work, and fostered liability concerns. One provider raised the concerns of medical liability and care responsibility when the patient is no longer under their direct supervision; all study providers shared this concern, with two providers in particular indicating the effect of remote monitoring on workflow:

‘*On the basis of reading and looking at [the patient’s] responses, I could have become extremely alarmed because [their] mood sounded like [they were] sinking in a hurry. … You know, it’s all subjective how they answer these things. But I would have to be on the phone every day checking for safety*’.

(PFG, P5)

‘*There’s liability concerns, for sure*’.

(PFG, P3)

‘*And that’s just not going to happen*’.

(PFG, P5)

‘*No, because it doesn’t fit into your existing workflow*’.

(PFG, P3)

## Discussion

What this qualitative study offers is an exploration of the providers experience with adopting and resisting the adoption of mobile health technology with the potential to disrupt workflow.

### Pre-visit

mHealth is often most effective when providers are able to access clinical information quickly and then use this information to focus on the issues at hand (Martinez, 2011). The app allowed providers to do just that. The app’s potential to improve care planning and self-management practices of complex care patients were witnessed during the initial on-boarding visit. Once health goals were set, providers had the ability to view patient-entered feedback at any time throughout the study, with the intention that these data sets could then be used to improve the care planning needs of their patients.

Providers appreciated the ability to view patient entered data and its potential to save valuable visit time; yet, when presented with the opportunity to streamline visit workflow, they failed to make effective use of the information available on the app before patient visits. Providers claimed the abundance of information available on the app overwhelmed them, maintaining the app could be perceived to be more useful if it provided graphic content that demonstrated the patient’s progress towards their goals. Interestingly, the app did provide graphic representations of health goals for quick orientation to patient reports. This result indicated perhaps more education is required for providers to fully benefit from the app, and more training is required to better understand the available features. Providers did not reject the new technology, but also were not willing to use the app to its full potential. Our findings are supported in the literature in which it has been shown that the relatively new wave of mobile technology, met with insufficient health informatics experts available to provide training and education support to healthcare professionals, can impact adoption (Yu *et al*., 2006; Peck, 2011).

### Patient visit

In addition to education and training, the perceived cost (time and effort) to incorporate the app into existing workflow was a concern for our primary care provider group. Provider reluctance was evidenced throughout the study. At study start, providers specifically questioned the impact of the app on their visit time, and then throughout the study questioned the value of data captured to improve care planning. When individuals are unready to change (ie, motivation readiness is not activated) their ability to undergo change behaviour practices to adapt to new technologies is unlikely (Lehman *et al.*, 2002).

When a mobile device is introduced into the provider–patient relationship, the device can be viewed as an unwelcome ‘third party’ and hinder provider–patient interaction by stealing attention away from the medical conversation and directing it to the device (Alsos *et al.*, 2012). However, due to the limited interaction with the app during patient appointments we found no indication of the app dominating provider–patient interaction. Although, in one instance a provider indicated pre-selecting patients for the app with whom they were familiar with, to minimize any impact to their workflow. Familiarity assumingly allowed the provider to rely on their past goal-setting experiences with the patient, and therefore reduced their need to access the app or alter their workflow during visits. Disruptive innovative technology solutions cannot be disruptive if users are unwilling to integrate solutions into their workflow.

The goal-setting process highlighted that integration is a key concern for technology adoption. The app forced providers to slightly shift their goal-setting process toward validated and reliable PROMIS tools from the traditional SMART goal template that they used and were familiar with. This change was viewed as unhelpful, with providers repeatedly indicating SMART goals are how they set goals. In fact the standardized outcome measures (PROMIS scales, HAQ, GAD-7, and PHQ-9) were altogether forgotten or ignored; providers did not use these tools to aid in shared decision-making and care planning activities with their patients. In addition, the app was unable to decrease documentation-related activities and instead increased the work required to complete visit reporting. Double work was created as interoperability between the app and the EMR was not possible. Regrettably, providers found the app too challenging to routinely fit into their existing visit workflows. It has been shown that providers may find mHealth technology disruptive to workflow when mobile technologies do not complement provider work habits, create additional work, or present unfavourable changes to familiar routines (Yu *et al*., 2006; Zheng *et al*., 2010). Further, failing to integrate mobile technologies with existing EMRs can effectively and negatively disrupt workflows (De Toledo *et al*., 2006).

### Post-visit

Although providers tended to shy away from incorporating the app into their workflows in the post-visit, patients on the other hand seemed more receptive of the technology. Morgan and Agee (2011) argue as patients become more engaged with mHealth solutions to improve their healthcare experience and access to providers and services, providers disengage. The limited provider–patient interaction with the app left many patients wondering how the app could be useful when provider-initiated feedback was rarely given. Providers offered little feedback during the monitoring period, with most discussing the monitoring with their patients at the mandatory patient visit at study’s end. In response to patient concerns, our providers suggested automatic feedback as an alternative way for their patients to feel empowered and engaged, while seemingly reducing their need to actively monitor or provide feedback, suggesting an attempt by providers to minimize their interaction with the app.

mHealth technology permits regular and long-term data monitoring preferable for the management of complex care patients and ideal for better-informed decision-making (McGrail *et al*., 2010; Leijdekkers and Gay, 2012). Regularly entered data have shown to improve the quality of diagnosis (Leijdekkers and Gay, 2012) and treatment, in part because providers are able to adjust patient care plans in accordance to patient-reported measures (McGrail *et al*., 2010). However, this study highlighted an important concern with remote patient monitoring – medical liability; who is responsible for care when the patient is no longer under direct medical supervision but continues to engage with their provider remotely? One provider frequently contacted their patient after reading alarming messages, but in each instance the patient was doing well. Patient-entered data were now viewed as subjective and disregarded. Recent literature states healthcare professionals find it increasingly difficult to monitor and provide appropriate care to the virtual patient (Lupton, 2006). Inaccurate data compromises reliability and inevitably effects remote patient monitoring and care quality, as providers may view patient entered data as suspicious, inaccurate, or altogether reject patient-reported measures as meaningful (Leijdekkers and Gay, 2012).

### Addressing resistive compliance

Our findings suggest strong evidence in support of Ward *et al*.’s (2008) notion of ‘resistive compliance’ amongst the providers in the study. At each point in the workflow we see evidence of general interest in adoption based on how providers spoke of the app, but actions of providers suggested resistance to full adoption, and a tendency towards maintaining the status quo in terms of workflows. Previous work has suggested that new system adoption needs to be supported through a multipronged approach which requires more than just adopting a new system but also requires organizational change (Lee *et al*., 2005). However, our approach was informed by a number of change management strategies including the use of strong organizational leadership, attention to provider workflows, and strategic planning of implementation.

Our findings suggest that perhaps the change management process can be tailored to address resistance at each stage of the workflow. At the pre-visit stage, education and knowledge about the full potential and capability of the technology was a barrier. Change management strategies that include a focus on how to incorporate needed information about the technology at the point of the pre-visit may address this barrier. During the patient visit, resistance was most related to interruptions in the workflow, and adoption may be greater if systems are better aligned. That being said, if the intention is to transform or modify the workflow or model of care, change management approaches which support distributed leadership from the frontline may be useful – allowing providers to feel like they are taking ownership over the new change (Best *et al*., 2013). Finally, resistance to ongoing monitoring during post-visit was strongly tied to liability issues, which may require change management processes at higher levels in the organization or even the health system to create safe environments in which technology can be adopted.

## Conclusions

Throughout our investigation we found a high level of provider resistance to change evidenced by consistent attempts on their part to shift the app towards completely fitting with workflow rather than adapting much of their behaviour. Even though providers saw potential in the app to assist in care planning and self-management for their complex care patients, they rarely engaged with the app, and mostly did so at study start and end, as was mandatory for study participation. What our study found was that new technologies cannot be disruptive in an environment that is resisting change. This is pivotal given the need for a shift in care delivery for this patient population (Tsasis and Bains, 2008; Lawn and Schoo, 2010). With that being said, to successfully integrate HIT within a FHT setting comprised of interdisciplinary healthcare professionals often adds an extra layer of complexity. Diverse roles, care responsibilities, and clinical needs make it increasingly difficult to accurately and consistently predict provider workflow demands (Pappas *et al*., 2002). If a digital ecosystem is to be created, and meaningful mHealth adoption is to occur, providers must be open to adapting to technology and adjusting work routines, especially if patient care can be improved. Disruptions to the status quo create opportunities for innovation. However, lessons learned from this qualitative study still, unfortunately, indicate much research is needed in understanding primary care provider workflows, responsibilities, and their resistance to adopting mHealth solutions. This report may be useful for researchers looking to understand provider resistance to adopting mHealth technologies in primary care practice workflows, to examine similarities, differences and key implementation issues across professional boundaries regarding the uptake of mobile technologies, and to improve applicability of our findings to a wider audience.

## Limitations

Although the study provided valuable insight into mHealth technologies and primary care provider workflow it is not without limitations. As with many mHealth studies generalizability is lacking. A small sample size allowed for setting specific observations. That being said, at this stage of the study, it was our intent to understand app usability at the local level, a single site, which justifies our qualitative study approach. Study timelines were tight resulting in short turnaround from provider training to study start, and additional time, education, and training beyond what was provided was likely required. In addition, system glitches affected optimal app function and may have negatively biased provider–patient interaction; however, as this phase of the ePRO development project focussed on usability, we anticipated system errors would occur. Furthermore, the choice to refrain from collecting greater provider demographic and socio-economic data did not allow for deeper investigation into provider metrics that may have effected app adoption. The choice to refrain from collecting provider data was consciously made, as a conversational approach was taken to build provider buy-in and relationships for future study phases.
